# Prevalence of active trachoma and associated risk factors among children of the pastoralist population in Madda Walabu rural district, Southeast Ethiopia: a community-based cross-sectional study

**DOI:** 10.1186/s12879-019-3992-5

**Published:** 2019-04-29

**Authors:** Kemal Kassim, Jeylan Kassim, Rameto Aman, Mohammedawel Abduku, Mekonnen Tegegne, Biniyam Sahiledengle

**Affiliations:** 1 The Fred Hollows Foundation Ethiopia, Bale-Robe, Ethiopia; 2 Department of Public Health, Madda Walabu University Goba Referral Hospital, Bale Goba, Ethiopia; 3 Department of Midwifery, Madda Walabu University Goba Referral Hospital, Bale Goba, Ethiopia

**Keywords:** Trachoma, Children, Pastoralist population, Bale zone, Ethiopia

## Abstract

**Background:**

In developing countries particularly in sub-Saharan Africa trachoma is still a public health concern. Ethiopia is the most affected of all and bears the highest burden of active trachoma. In spite of this, the prevalence of active trachoma among the pastoralist population in Ethiopia not yet disclosed. The aim of this study was to determine the prevalence of active trachoma and associated risk factors among children in a pastoralist population in Madda Walabu rural district, Ethiopia.

**Methods:**

A community-based cross-sectional study was conducted among children in a pastoralist population in Madda Walabu rural district, from May 1 to 30, 2017. A systematic sampling technique was employed to select 409 children’s. Simplified WHO classification scheme was used to assess trachoma. Descriptive and logistic regression analyses were performed.

**Results:**

A total of 406 children aged 1–9 years have participated, 89 (22%) [95%CI: 18.0–25.6%] were positive for active trachoma. Of these cases, 75(84%) had TI alone in one or both eyes, 14(16%) had TF alone in one or both eyes, and none of the children had both TI and TF. The odds of having active trachoma among children from households using river/ponds, unprotected well/spring and rainwater as their source of drinking water were higher than those from households using water from piped or public tap water (AOR:13,95%CI: 2.9, 58.2), (AOR: 6.1, 95%CI:1.0,36.5) and (AOR: 4.8, 95%CI:1.3,17.8) respectively. Children’s from households that lacked a latrine (AOR: 2.5, 95% CI: 1.8, 5.3), children who did not wash their face by using soap (AOR: 4.3, 95% CI: 1.8, 10.6) and children from households within 16–30 min of water source (AOR: 8.7, 95% CI: 2.20, 34.2) were higher odds of having active trachoma.

**Conclusions:**

The findings of this study revealed that close to one-quarter of the total children screened for trachoma were positive for the disease. The finding implies that trachoma is still a major concern among children of the pastoralist community which demands further attention of the district health office. Again, intervention with the A, F and E components of SAFE strategy is strongly recommended.

**Electronic supplementary material:**

The online version of this article (10.1186/s12879-019-3992-5) contains supplementary material, which is available to authorized users.

## Background

Trachoma is a disease caused by the bacterium C. *trachomatis*. According to the World Health Organization (WHO) simplified trachoma grading system, redness, and irritation, with follicles on the tarsal conjunctiva, meet the definition of trachomatous inflammation– follicular (TF) [1]. The infection commonly spreads from person to person through direct contact [1, 2]. According to the recent systematic review estimate, trachoma accounts for 0.45 million blindness and 1.4 million vision impartment [2]. Moreover, it is considered as a public health problem in more than 50 countries, with an estimated 158 million people at risk of blindness [1, 3].

Blinding trachoma also considered as a neglected tropical disease and earmarked for global elimination by the year 2020, using the advocated World Health Organization (WHO) recommended a SAFE strategy. This involves Surgery for trichiasis, Antibiotics for infection, Facial cleanliness to limit transmission, and Environmental improvement [4, 5].

In Sub-Saharan Africa, trachoma is a leading cause of preventable blindness [4]. And, Ethiopia is the most affected of all and bears the highest burden of active trachoma [3, 6–9]. A study conducted by the Global Trachoma Mapping Project from December 2012 to July 2014 in the Oromia regional state of Ethiopia reported the overall age-adjusted TF prevalence in 1–9-year-olds was 23.4% [7]. Studies conducted elsewhere in Ethiopia also recorded a high prevalence of trachoma; in Gondar Zuria district the prevalence of active trachoma was 12.1% [10], Leku town (southern Ethiopia) the overall prevalence of active trachoma found to be 11.0% (Trachomatous inflammation- Follicular 9.8% and Intense 1.2%) [11]. In the same way, a high burden of active trachoma repeatedly reported from all corner of the country [11–17]. And in many rural parts of Ethiopia, trachoma continues to be rampant. For example, in the north and south Wollo zones of Amhara region the overall prevalence of active trachoma among children aged 1–9 was 21.6% (18% of TF cases and 4.7% were TI cases) [16]. In Gazegibela district of Wagehemra zone, among children aged 1–9 years 52.4% were positive for active trachoma. Of these cases, 49.1% were trachomatous inflammation-follicular and 3.3% were trachomatous inflammation-intense [17].

Despite these extensive studies data on the prevalence of active trachoma among the pastoralist population still limited. Moreover, none of the previously conducted studies assesses the prevalence of active trachoma among the pastoralist population in southeast Ethiopia expect one study which a baseline survey conducted in 2013 by Global Trachoma Mapping Project (GTMP), which reported the prevalence of TF in the district was 38.8% [7]. Hence, reliable population-based prevalence data in such community is very important for planning and implementation of effective trachoma control programs, for the country like Ethiopia where trachoma ranks the first in the list of high burden countries. Furthermore, the current study is crucial to evaluate the ongoing intervention. Therefore, the objective of this study was to determine the prevalence of active trachoma and associated risk factors among children in the pastoralist population in Madda Walabu rural district, southeast Ethiopia.

## Methods

### Study design and settings

A community-based cross-sectional study was employed among children age 1–9 years of the pastoralist populations in Madda Walabu rural district, Oromia Regional State, Bale Zone, Southeast Ethiopia from May 1 to 30, 2017. Oromia is the largest of the nine regions of Ethiopia by both landmass and number of residents, according to the 2005–2006 national survey of blindness; low vision and trachoma estimated the region-level prevalence of TF among children 1–9 years of age to be 24.5% [9]. In addition, the recent population-based prevalence surveys conducted with the Global Trachoma Mapping Project baseline trachoma map in Oromia reported that both active trachoma and trichiasis are highly prevalent in Oromia, constituting a significant public health problem for the region [7]. According to Global Trachoma Mapping Project baseline trachoma report, the TF prevalence among children aged 1–9 years ranged from a low of 1.1% in Gaji woreda of West Wellega to a high of 48.7% in the Horo Guduru zone woredas [7]. According to 2013 GTMP figures and the trachoma atlas report, Madda Walabu was known for endemic trachoma [18].

Madda Walabu is one among the nine pastoralist districts of the Bale Zone. Madda Walabu is mainly characterized by cattle livestock farmer population. It is located 630 Km from Addis Ababa (the capital city of Ethiopia). It has 22 kebeles (sub-districts) with a total population of 127,682 according to Bale Zone health office 2016/2017 population projection. In the district, there are one primary hospital, 6 health centers, and 20 health posts. There is also one Integrated Eye Care Worker (IECW) service.

### Sample size determination

The sample size was determined using a single population proportion formula by considering the following assumptions: *p* = 41.3% prevalence of active trachoma in children 1–9 years in Oromia regional state [9], 95% confidence interval (CI), and a 5% margin of error and considering 10% non-response rate, the final sample size was 409 children.

### Sampling procedure

A systematic sampling technique was employed to select children age 1–9 years. From 22 kebeles (sub-districts) of Madda Walabu district, six rural sub-districts were selected using a simple random sampling technique. Consequently, Waltae Burra, Ware, Ela Bidire, Berisa, Oda Boji and Oda sub-districts were selected. After sampling frame preparation, that contains lists of households with children age 1–9 years old from health extension house to house visit routine program list, the calculated sample sizes were allocated proportionally to size for each selected sub-districts. Then, a systematic sampling technique was applied to select households that had at least one 1–9 years old child. For households that had more than one child aged 1–9 years, a single child was selected by using the lottery method.

### Data collection procedures

A pre-tested structured questionnaire was used to collect the data by trained two data collectors (who record the data) (Additional file 1). Following the interview eye examination of both eyes of the selected child was done using binocular loupe of magnification convergent (2.5x) by three senior Integrated Eye Care Workers (IECWs) who had been adequately trained, well experienced and attended the standardized 5-day training using GTMP training [19]. Each eye was examined separately by careful inspection of eyelashes, cornea, limbus, eversion of the upper lid and inspection of the tarsal conjunctiva and results of the examination were registered. The steps of eye examination and trachoma grading strictly followed the WHO simplified grading system [4]. To prevent cross infection between successive participants, the necessary hygienic measures were also taken through cleaning hands with alcohol-based hand gel after each examination.

### Study variables

Dependent variable: presence of active trachoma (yes/no).

Independent variables: sex (male, female), age (1–4 years, 5–9 years), parent-reported frequency of face washing (occasionally, once per day and more than once per day), parent-reported use of soap when face last washed (yes, no), source of water (piped water/public tap, river/pond, unprotected well/spring, rainwater, protected well/spring), time to collect water (< 15 min’ walk, 16–30 min’ walk, 31–60 min’ walk and > 60 min’ walk), presence of latrine (yes, no), heard about trachoma (yes, no).

### Operational definition

Active trachoma: the standard WHO simplified grading system was used to identify active trachoma.

Trachomatous inflammation follicular (TF): has been suggested by WHO as the key indicator for assessing active trachoma and it was defined as the presence of five or more follicles in the upper tarsal conjunctiva (follicles must be at least 0.5 mm in diameter).

Trachomatous inflammation-intense (TI): pronounced inflammatory thickening of the tarsal conjunctiva that obscures more than half of the normal deep tarsal vessels. In this study, the outcome variable was overall active trachoma.

### Data processing and analysis

Data were entered into Epi Info™ version 3.5.1software and analyzed using Statistical Package for Social Sciences (SPSS) version 20.0 (IBM Corporation 2012) statistical software. A descriptive analysis was computed to present important variables and to describe the prevalence of active trachoma. Bivariate and multivariable logistic regression analysis was computed. Adjusted odds ratio (AOR) with 95% confidence interval (CI) was used to determine the strength of association and a *p*-value less than 0.05 was considered as a cut-off point to declare statistical significance.

### Data quality

The questionnaire was pre-tested in the nearby non-study area (Harena Buluk district) prior to the actual data collection period and the necessary corrections were made. In addition, a two-day extensive training was given for data collectors and field supervisors prior to the commencement of the data collection. Moreover, eye examination was carried out by senior Integrated Eye Care Workers (IECWs), they were also trained for 2 days’ on the use of the WHO simplified grading system, and data collection procedures. Data completeness and consistency was checked daily bases by supervisors and investigators.

## Results

### Socio-demographic characteristics of the respondents

A total of 406 children aged 1–9 years have participated in the study. Of them, 215 (53%) were males. Mean age of the study participants was 4.8 with a standard deviation (SD) of 2.37 years. Most (94%) of households the heads were farmers by occupation. In the study area, the mean family size among the study participants was 6.5 (SD ± 2.63) persons (Table 1).

**Table 1:** Socio-demographic characteristics of study participants in Madda Walabu district, Southeast Ethiopia, May 2017 (*n* = 406)

Variables	Frequency	Percentage (%)
Sex of the household head		
Male	338	83.3
Female	68	16.7
Educational status of the household head		
Illiterate	273	67.2
Can read & write	74	18.2
Elementary school	54	13.3
High school & above	5	1.2
Occupation of the household head		
Farmer	382	94.1
Daily laborer	4	1.0
Merchant	15	3.7
Employed	5	1.2
Sex of child		
Male	215	53.0
Female	191	47.0
Age of child		
1–4 years	200	49.3
5–9 years	206	51.7
Educational status of the child		
Too young	260	64.0
Not enrolled	58	14.3
Dropped out of school	11	2.7
Attending school	77	19.0

### Environmental and behavioral related variables

Almost half (45.6%) of the households get their water supply from unprotected sources. In addition, more than half (54.4%) of the respondent get water traveling for more than 15 min’ walk. Half of the households (51.5%) dispose of their domestic wastes in open field. About two-thirds (68.7%) of the households have pit latrine (Table 2).

**Table 2:** Environmental and other factors associated with the occurrence of trachoma in Madda Walabu district, Southeast Ethiopia, May 2017 (*n* = 406)

Variables	Frequency	Percentage (%)
Source of drinking water		
Piped water/public tap	176	43.3
Unprotected well/spring	105	25.9
Rainwater	45	11.1
Surface water (River/pond)	43	10.6
Protected well/spring	37	9.1
Time to fetch water		
< 15 min’ walk	185	45.6
16–30 min’ walk	83	20.4
31–60 min’ walk	72	17.7
> 60 min’ walk	66	16.3
Presence of latrine		
Yes	279	68.7
No	127	31.3
Hand washing facility near latrine (*n* = 279)		
Yes	57	20.4
No	222	79.6
The solid waste disposal site		
Open field	209	51.5
Burning/burying	177	43.6
In uncovered pit	17	4.2
In covered pit	3	0.7
A parent reported the frequency of face washing		
More than once per day	179	44.1
At least once per day	114	28.1
Only occasionally	113	27.8
A parent reported the use of soap when face last washed		
Yes	178	43.8
No	228	56.2
Heard about trachoma		
Yes	355	87.4
No	51	12.6
Presences of eye drainage		
Yes	94	23.2
No	312	76.8
Nasal discharge		
Yes	126	31.0
No	280	69.0
Presence of fly on the face		
Yes	154	37.9
No	252	62.1

### Prevalence of active trachoma

The overall prevalence of active trachoma was 21.9% (95%CI:18.0–25.6%). Of these cases, TF and TI alone account 18.5 and 3.4%, respectively. And in our assessment, we did not find children with both TF and TI.

### Factors associated with the prevalence of active trachoma

In the bivariate analysis, children from households using river/ponds, unprotected well/spring and rainwater as their source of drinking water, children from households that lacked a latrine, children from households within 16–30 min of water source, children who did not washed their face by using soap and who wash their face occasionally and children’s from households that did not heard about trachoma were factors found to be associated with the presence of active trachoma.

In multivariable logistic regression analyses, the odds of having active trachoma among children from households using river/ponds, unprotected well/spring and rainwater as their source of drinking water were 13, 6.1 and 4.8 times higher than those from households using water from piped or public tap water (AOR: 13, 95%CI: 2.9, 58.2), (AOR: 6.1, 95%CI:1.0,36.5) and (AOR: 4.8, 95%CI:1.3,17.8) respectively. The odds of having active trachoma among children from households that lacked a latrine were 2.5 times higher than their counterparts (AOR: 2.5, 95% CI: 1.8, 5.3). In addition, the odds of having active trachoma among children from households within 16–30 min of water source were almost nine times higher than those from households who fetch less than 15 min (AOR: 8.7, 95% CI: 2.20, 34.2). Moreover, the odds of having active trachoma among children who did not wash their face by using soap were 4.3 times higher compared to those who washed their face by using soap (AOR: 4.3, 95% CI: 1.8, 10.6) (Table 3).

**Table 3:** Factors associated with the prevalence of active trachoma among children age 1–9 years at Madda Walabu district, Southeast Ethiopia, May 2017

Variables	Trachoma		Crude OR (95%CI)	Adjusted OR (95%CI)
	Yes (*n* = 89)	No (*n* = 317)		
Source of drinking water				
Piped water/public tap	7	169	1	1
Surface water (River/pond)	5	38	87.5 (29.5259.8)*	13 (2.9, 58.2)**
Unprotected well/spring	18	87	27.6 (8.2,93.1)*	6.1 (1.0,36.5)**
Rain water	30	15	17.5 (6.9,44.5)*	4.8 (1.3,17.8)**
Protected well/spring	29	8	1.8 (0.67,4.9)	1.7 (0.51,5.5)
Sex of the child				
Male	42	173	0.74 (0.48,1.2)	1.4 (0.68,2.95)
Female	47	144	1	1
Time to fetch water				
< 15 min’ walk	6	179	1	1
16–30 min’ walk	9	74	85.9 (32.2229.8)*	8.7 (2.20,34.2)**
31–60 min’ walk	25	47	23.7 (9.80,57.4)*	2.6 (0.62,11.1)
> 60 min’ walk	49	17	5.4 (2.6,11.3)*	2.4 (0.85,6.9)
Presence of latrine				
Yes	34	245	1	1
No	55	72	5.5 (3.30,9.10)*	2.5 (1.80,5.30)**
A parent reported the use of soap when face last washed				
Yes	9	169	1	1
No	80	148	10.2 (4.90,20.9)*	4.3 (1.80,10.60)**
A parent reported face washing frequency				
Occasionally	45	68	1	1
Once per day	31	83	0.12 (0.06,0.23)*	0.41 (0.15,1.11)
More than once per day	13	166	0.21 (0.10,0.42)*	0.40 (0.15,1.10)
Heard about trachoma				
Yes	70	285	1	1
No	19	32	2.42 (1.29,4.52)*	0.72 (0.27,1.90)

*OR* Odds Ratio *(*P* < 0.05) crude, ** (*p* < 0.05) adjusted

## Discussion

The prevalence of active trachoma among children aged 1–9 in the study population was found to be 21.9% (18.5% were TF cases and 3.4% were TI), which is higher than the WHO trachoma elimination target (a prevalence of active trachoma (grade TF) in children aged 1–9 years of < 5%) [13]. The current finding was significantly lower than the prevalence of TF in children aged 1–9 years by Global Trachoma Mapping Project in the year 2012–2014, which reported the prevalence of TF was 38.8% in Berbere, Dolo Mena, Gura Damole, Harena Buluk, Meda Welabu districts [7]. The low prevalence of TF observed in this study may be due to the woredas mass distribution of azithromycin, together with the implementation of the F and E components of the SAFE strategy in Madda Walabu district. Moreover, the other possible explanation for this variation may be due to the time gap and sample size dissimilarity. On one hand, the current high prevalence of active trachoma almost in agreement with a study conducted in a similar setting in Oromia regional state (Ethiopia), 23.4% [7].

On the other hand, the prevalence of active trachoma in this study was lower than a study report from prevalence Gazegibela district, Amhara region, which reported 52.4% [17], and a report from Dalocha district, central Ethiopia reported 51.5% prevalence [20]. This difference might be due to infrastructure and health service coverage difference, in addition to Gazegibela district, which is repeatedly drought affected and one of the food insecure districts of the Amhara region. The prevalence of active trachoma in our study is also a little lower than the prevalence rate reported from Malawi 25.1% [21]. The present finding, TF prevalence > 10% among children 1–9 years old in this district still a concern and mass distribution of azithromycin, together with the implementation of the F and E components of the SAFE strategy should be strengthened.

In multivariable logistic regression analysis, a significant association was observed between the presence of active trachoma and source of drinking water, time to collect water, households that lacked a latrine, and parent-reported the use of soap when face last washed. Unlike the findings from the north and south Wollo Zones of Amhara region, this study finding a significant association between the source of water and the presence of active trachoma [16]. In line with this finding, a study from Gazegibela district (north Ethiopia) reported the source of water supply has been significantly associated with the occurrence of active trachoma among children [17].

Children from families that spend up to 30 min walking to a water source were more likely to have active trachoma than those that spend less than 15 min. In agreement with this finding studies from the north and south Wollo Zones of Amhara region [16], and from Ankober (Ethiopia) [22] documented the importance of time to fetch water on the prevalence of trachoma. Similar findings have also been documented in Tanzania [23].

The odds of having active trachoma among children’s from households that lacked a latrine were almost threefold times higher than their counterparts. In support of this, other studies also reported a similar finding [10, 15, 17, 24].

The use of soap for face washing has been important factors in the prevalence of active trachoma. The odds of having trachoma among children who did not use soap when face last washed had four times higher compared to those use soap during washing. A comparable finding also reported from other previously conducted studies [10, 11]. Hence, improving community hygiene practice through awareness creation activities is very crucial.

The study has some major limitations to the estimations of household time to fetched water were merely based on the respondents’ response, which may be uncertain. In order to minimize the errors, the study took the average value. In addition, the self-reported practice towards some interviewer questions such as parent-reported use of soap when face last washed and parent-reported frequency of face washing practice may be uncertain due to social desirability bias. Since there are limited studies conducted in such a pastoralist community the study compared the prevalence of active trachoma with other community, for this reason, readers must take caution while reading the article. One additional limitation of this study is that we did not use direct observation to record the water or latrine uses, that limits our finding to validate self-reported practice.

## Conclusion

In the study area of Madda Walabu district the prevalence of active trachoma still highly prevalent. Children from households that lacked a latrine and using river/ponds, unprotected well/spring and rainwater as their source of drinking water were higher odds of developing active trachoma. In addition, children who did not wash their face by using soap were higher odds of developing active trachoma. The finding implies that trachoma is a major concern among children of the pastoralist community which demands further attention of the district health office, regional government and different stakeholders, who designing and implementing trachoma elimination programs. Again, intervention with the A, F and E components of SAFE strategy is strongly recommended. In addition, screening adults in the community for trichiasis would be a good idea.

## Additional File

Additional file 1: Data collection tool. (PDF 274 kb)

## Abbreviations

AOR: Adjusted odds ratio
CI: Confidence interval
GTMP: Global Trachoma Mapping Project
IECW: Integrated Eye Care Worker
OR: Odds ratio
SAFE: Surgery for trichiasis, Antibiotics for infection, Facial cleanliness to stop transmission, and Environmental improvement
SD: Standard deviation
SPSS: Statistical Package for Social Sciences
TF: Trachomatous inflammation-follicular
TI: Trachomatous inflammation-intense
WHO: World Health Organization
