# Supplementary material for: Prevalence of active trachoma and associated risk factors among children of the pastoralist population in Madda Walabu rural district, Southeast Ethiopia: a community-based cross-sectional study
Source: BMC Infect Dis. 2019 Apr 29;19:353. doi: 10.1186/s12879-019-3992-5 (PMC6489250; doi:10.1186/s12879-019-3992-5)
Supplement: Supplementary file 1 — Data collection tool. (PDF 274 kb) [file 12879_2019_3992_MOESM1_ESM.pdf]

Data Collection Tool

<table>
<tr><th>No.</th><th>Questions</th><th>Answers (Coding Category)</th></tr>
<tr><td>111</td><td>Unique Questionnaire No.</td><td></td></tr>
<tr><td>112</td><td>Age of head of household in years</td><td>|___|___|</td></tr>
<tr><td>113</td><td>Sex of head of household</td><td>1. Male
2. Female</td></tr>
<tr><td>114</td><td>What is the highest level of school you have attended?</td><td>1. Illiterate
2. Can read and write
3. Elementary school
4. High school & above</td></tr>
<tr><td>115</td><td>What is the main occupation of the head of the household?</td><td>1. Farmer
2. Daily laborer
3. Merchant
4. Government employee
5. Others, specify______________</td></tr>
<tr><td>116</td><td>How many people live in this household?</td><td>|___|</td></tr>
<tr><td>117</td><td>Number of children <10 years in the household</td><td>|___|</td></tr>
<tr><td>118</td><td>Age of selected child in years</td><td>|___|</td></tr>
<tr><td>119</td><td>Sex of selected child</td><td>1. Male
2. Female</td></tr>
<tr><td>120</td><td>Educational status of child</td><td>1. Too young to go to school (pre-school child)
2. Not enrolled (not joined school) yet
3. Dropped out of school
4. Attending school</td></tr>
<tr><td>121</td><td>If the selected child is in school or drop out of school, what is the highest grade completed?</td><td>|___|</td></tr>
<tr><td>122</td><td>Number of living rooms in the house</td><td>|___|</td></tr>
<tr><td>123</td><td>What is the main source of water for domestic use of members of your household?</td><td>1. Piped water/public tap
2. Protected well/spring
3. Unprotected well/spring
4. Rain water collection
5. Surface water (River/pond)</td></tr>
<tr><td>124</td><td>Time to main source of water for domestic use (round trip in minutes)?</td><td>1. <15 minutes
2. 16-30 minutes
3. 31-60 minutes
4. >60 minutes</td></tr>
<tr><td>125</td><td>Amount of water used per day (estimate)</td><td>1. Greater than 80 liters</td></tr>
</table>

		2. 60–80 liters<br>3. 20–40 liters<br>4. Less than 20 liters
126	Does your household have access to a latrine? (Interviewer needs to verify presence of pit latrine)	1. Yes<br>2. No
127	Ask to see the latrine/toilet.<br>Observation: What kind of toilet facility does the household use?	1. Pit latrine<br>2. No facility, they use bush or field<br>3. Other (specify)______________
128	Latrine use (who uses latrine in the household?)	1. Only adults<br>2. Both adults and children<br>3. Not regularly /consistently used
129	Observation: Is there a hand washing facility within 15 meters of the latrine/toilet?	1. Yes<br>2. No<br>3. Not applicable (no latrine)
130	Observation: At the time of the visit, is water available at the hand washing facility?	1. Yes<br>2. No<br>3. Not applicable (no hand washing facility
131	Observation: At the time of the visit, is soap or ash available at the handwashing facility?	0. Yes<br>1. No<br>2. Not applicable (no hand washing facility)
133	Where do you dispose of your garbage? (Interviewer needs to verify presence of pit)	1. In open field<br>2. Burning/Burying<br>3. In covered pit<br>4. In uncovered pit<br>5. Other, specify________________
134	How often does this child wash his/her face? (parent reported)	1. Only occasionally<br>2. At least once per day<br>3. More than once per day
135	Soap use when face last washed (parent reported)	1. Yes<br>2. No
136	Do you have any animals (cattle, sheep, goats, camels)?	1. Yes<br>2. No
137	Do you keep your animals (cattle, sheep, goats, camels) in the house you are living in? or within 20 meters of the houses?	1. No, keep separately<br>2. Yes, only at night<br>3. Yes, only during the day<br>4. Yes, both at night & during the day

138	Is there animal dung in the compound? (Observe)	1. Yes<br>2. No
139	Have you ever heard health information on trachoma?	1. Yes<br>2. No
140	Where did you hear the trachoma information?	1. School/teachers<br>2. Trachoma volunteers<br>3. Health extension worker<br>4. Mass media (TV, radio, etc)<br>5. Health facility<br>6. Community gatherings<br>7. Other, specify_______________
140	Distance of the nearby health 1enter on foot	1. <15 minutes<br>2. 16-30 minutes<br>3. 31-60 minutes<br>4. >60 minutes

Part II: Facial observation and eye examination

**S.No.**	**Variable Description/Question**	**Answer/Code**
201	Is there ocular discharge in the child's face?	1. Yes<br>2. No
202	Is there a nasal discharge in the child's face?	1. Yes<br>2. No
203	Is there a fly on the child's face, within 3 seconds of observation?	1. Yes<br>2. No
204	Is there sign of active trachoma on eye examination?	1. Yes<br>2. No
205	If Q 204 Yes, specify the stage of trachoma	1. TF<br>2. TI<br>3. TF & TI<br>4. TT

Thank you for your participation.
